# Retracted: Production of Gymnemic Acid Depends on Medium, Explants, PGRs, Color Lights, Temperature, Photoperiod, and Sucrose Sources in Batch Culture of *Gymnema sylvestre*

**DOI:** 10.1155/2019/9690578

**Published:** 2019-11-20

**Authors:** The Scientific World Journal

The Scientific World Journal has retracted the article titled “Production of Gymnemic Acid Depends on Medium, Explants, PGRs, Color Lights, Temperature, Photoperiod, and Sucrose Sources in Batch Culture of* Gymnema sylvestre*” [1]. As raised on PubPeer, figure panels are duplicated within the article and many also appear in the authors' other publications in* Spanish Journal of Agricultural Research* [2], Protocols for In Vitro Cultures and Secondary Metabolite Analysis of Aromatic and Medicinal Plants [3],* AgroFOOD industry hi-tech* [4],* Acta Chromatographica* [5], 2014 4th International Conference on Biotechnology and Environment Management, IPCBEE [6], and* KMITL Science and Technology Journal* [7]. The authors did not provide the original images or the raw data, and the Editorial Board found their response was not satisfactory and recommended retraction.

Details of the main concerns, in which images are reused though they do not represent the same experiments, are as follows:

(i) Figures 1(b), 1(g), and 1(k) in this article appear to be the same as Figures 2F, 2H, and 2C, respectively, in A. B. A. Ahmed, R. Pallela, A. S. Rao, M. V. Rao, R. Mat Taha, “Optimized Conditions for Callus Induction, Plant Regeneration and Alkaloids Accumulation in Stem and Shoot Tip Explants of Phyla nodiflora,”* Spanish Journal of Agricultural Research* 2011 9(4), 1262-1270 [2]. However, the articles study different species,* Gymnema* and* Phyla*.

(ii) Figure 1(i) in this article appears to be the same as Figure 1(m), though flipped horizontally.

(iii) Figure 1(j) in this article appears to be the same as Figure 4(i).

Additionally, the following figures and results may represent the same experiments, but the earlier articles were not cited and the reuse was not indicated:

(i) Figures 5(a)–5(d) in this article appear to be the same as Figures 2a–2d, respectively, in Abdul Bakrudeen Ali Ahmed, Adhikarla Suryanarayana Rao, Mandali Venkateswara Rao, “In Vitro Production of Gymnemic Acid from* Gymnema sylvestre* (Retz) R. Br. Ex Roemer and Schultes through Callus Culture Under Abiotic Stress Conditions,” Protocols for In Vitro Cultures and Secondary Metabolite Analysis of Aromatic and Medicinal Plants, 2009, Volume 547 of the series Methods in Molecular Biology pp 93-105 [3]. In addition, the dry weights of callus biomass in Figures 2(a), 2(c), and 2(e) in this article for B5, SH, and MS media, respectively, appear to be the same as those in Table 1 in the other article.

(ii) Figures 1(i), 4(a)–4(d), 5(a), and 5(d) in this article appear to be the same as Figures 2b, 2a–2d, 5, and 6, respectively, in A. Bakrudeen Ali Ahmed, A.S. Rao, M.V. Rao, R.M. Taha, “Different Wavelengths Light to Induce Physiological Changes Callus for the Biosynthesis of Gymnemic Acid in* Gymnema sylvestre*,” AgroFOOD industry hi-tech - May/June 2012 - vol 23 n 3, pp. 31-34 [4].

(iii) Figures 1(i), 1(j), 4(a), 4(h), 4(j), 4(l), 5(a), and 5(b) in this article appear to be the same as Figures 1c, 1d, 1b, 1f, 1e, 1g, 4b, and 4e, respectively, in A.B.A. Ahmed, A.S. Rao, M.V. Rao, R.M. Taha, “HPTLC/HPLC and Gravimetric Methodology for the Identification and Quantification of Gymnemic Acid from* Gymnema sylvestre* Methanolic Extracts,”* Acta Chromatographica* 25(2013)2, 339–361, 0231–2522 [5].

(iv) Figures 4(a)–4(d), 5(a), 5(b), and 5(d) in this article appear to be the same as Figures 1a–1d, 2, 3, and 4, respectively, in Bakrudeen Ali Ahmed Abdul, Rao, M.V, Rao, A.S, Rosna Mat Taha, “Optimization of Gymnemic Acid Production with Anti-Diabetic Studies and Regeneration of Langerhans Cells from* Gymnema sylvestre*,” 2014 4th International Conference on Biotechnology and Environment Management, IPCBEE vol.75 (2014) [6].

(v) Figures 1(a) and 1(d) in this article appear to be the same as Figures 1a and 1d (right), respectively, in Abdul Bakrudeen Ali Ahmed, Adhikarla Suryanarayana Rao, Mandali Venkateswara Rao, “Somatic Embryogenesis and Plant Regeneration from Cell Suspension Culture of* Gymnema sylvestre* (Retz) R. Br. Ex Roemer & Schultes,”* KMITL Science and Technology Journal* Vol. 9 No. 1 Jan. - Jun. 2009, pp. 18-26 [7].
